# Supplementary material for: Are carbon emissions trading and green financial instruments synergistic? -Comprehensive quantitative research based on content analysis
Source: PLoS One. 2024 Mar 7;19(3):e0298601. doi: 10.1371/journal.pone.0298601 (PMC10919601; doi:10.1371/journal.pone.0298601)
Supplement: S1 Data — (ZIP) [file pone.0298601.s001.zip › Data/地级市Z_F工作报告.zip!/░▓╗╒/╦▐╓▌╩╨/└┤╫╘╣1⁄2╓┌║┼í╛┬φ┐╦╩2╛▌═°í┐_2011.docx]

——2011年1月11日在宿州市第三届人民代表大会第三次会议上

代市长   张曙光

各位代表：

  现在，我代表市人民政府，向大会报告工作，请予审议，并请市政协委员和其他列席人员提出意见。

  一、团结拼搏，2010年及“十一五”成就显著

  刚刚过去的一年，全市人民在市委的坚强领导下，全面贯彻科学发展观，紧紧围绕“双千亿、双翻番”目标，克难奋进，扎实工作，圆满完成了市三届人大二次会议确定的各项任务。预计全市生产总值突破600亿元，增长13%左右，人均GDP达到1500美元；财政总收入43.2亿元，增长32.5%；固定资产投资400亿元，增长35%；社会消费品零售总额194亿元，增长18%以上；城镇居民人均可支配收入14600元，农民人均纯收入4500元，分别增长12%、10%左右。

  第一，扩大有效投入，发展基础不断夯实。全力抓好重大项目建设，完成工业投资160亿元，“861”项目投资完成比例居全省第一。钱营孜煤矿、百丽鞋业、天瑞水泥、德利服饰等项目建成投产，蕲县船闸、汉泰化工、金黄庄和龙王庙煤矿建设加快，海螺水泥、国电宿州热电联产即将开工。京沪高铁完成全线铺轨，泗许高速一期建成通车，206国道等三个世行项目交工验收，宿淮铁路进展顺利，徐明高速、泗许高速二期、济祁高速公路开工建设。220千伏桥开关站、虹乡输变电投入运行。

  第二，调整经济结构，运行质量不断提升。实施工业五年扩张战略，规模以上工业增加值完成210亿元，增长25%以上。规模以上企业达到893户，其中超亿元企业140户。节能减排目标全面实现。粮食连续7年丰收，总产达389万吨。市级以上产业化龙头企业225家，农产品加工产值突破300亿元。林木产业、农机化综合水平保持全省领先。农田水利三年恢复性工程成效显著，重要河道堤防防洪排涝标准进一步提高。认真落实汽车、家电下乡和以旧换新政策，消费需求快速增长。进出口总额完成1.4亿美元，增长64%。

  第三，实施城镇扩容，城乡环境不断改善。深入开展城市建设管理年活动，中心城市建设步伐加快。城市总规等四个层面规划编制有序推进。投资65亿元，实施8大类165项重点工程，拂晓南路等11条道路建成通车，三角洲生态公园等3项景观工程、市博物馆等8项公益设施圆满竣工，市民文化活动中心四项工程建成启用。争创省级文明城市活动成效显著，城市环境和品位明显提升。县城建设加快，中心镇总体规划编制完成率100%，村镇建设完成投资25亿元。

  第四，深化改革开放，经济活力不断增强。加快非公经济发展，新增私营企业1802家。全面完成市县两级政府机构改革，深入推进农村综合改革、基层医药卫生和文化体制改革，农村土地整治示范建设开局良好，市演艺集团挂牌成立。加大金融领域创新，获准筹建总分行制村镇银行1家，金融机构贷款余额比年初净增51.5亿元。主动承接产业转移，开展招商百日竞赛，到位内资300亿元，增长40%；实际利用外资1.4亿美元，增长25%。成功举办第四届灵璧石国际文化节、中国果蔬加工砀山论坛。全面开展与央企、省企、知名民企对接，与马鞍山市合作不断深化。6个省级经济开发区转型升级步伐加快，14个新型乡村工业园入驻企业105家。

  第五，构建和谐宿州，人民生活不断提高。实施33项民生工程，投入各类资金27.4亿元。建成廉租住房3124套、农家书屋181个、农村留守儿童之家787个、农民体育健身工程209个，实施900例贫困白内障患者复明手术，新农合覆盖率达94.8%。新增城镇就业3.4万人，登记失业率控制在4%。城乡低保实行动态管理，“五险”扩面新增11.4万人，100万农村居民参加新型社会养老保险试点，7.7万被征地农民参加养老保险，城镇居民医保参保率达90%以上。新建敬老院46所、农村户用沼气1万户，解决23万农村人口饮水安全问题，所有行政村通达公路。扎实推进平安宿州建设，及时化解各类矛盾，安全生产形势总体向好，道路交通、消防形势较为平稳，食品药品监管规范有序，社会保持和谐稳定。

  第六，统筹发展各项事业，社会建设不断加强。实施技术创新工程，认定国家级高新技术企业1家、高新技术产品19项。免除学杂费3.2亿元，完成中小学校舍安全加固改造工程81万平方米，西北新区中学开工建设。基层医疗卫生服务体系进一步加强，8个乡镇卫生院、615个村卫生室竣工使用，基本公共卫生服务均等化逐步推进。荣获全国无偿献血先进市称号。成功举办第五届文化艺术节，砀山唢呐等3个项目入选第三批国家级非物质文化遗产保护名录。我市健儿在广州亚运会、亚残会上荣获2枚金牌。低生育水平继续稳定，出生人口性别比偏高势头得到有效遏制。“五五普法”通过验收。第六次全国人口普查工作顺利推进。国防动员、人民防空和双拥优抚工作取得新成绩，广播电视、外事侨务、民族宗教、应急管理、物价、对台、档案、地方志工作不断加强，扶贫开发、气象、防震减灾工作扎实推进，工会、共青团、妇女儿童、残疾人事业实现新进展。

  过去的一年，我们注重加强政府自身建设，自觉接受人大法律监督、政协民主监督和社会舆论监督，注意听取各方意见和建议，按期办结人大议案3件、代表建议98件、政协委员提案277件。深化行政审批制度改革，基建审批平台建设稳步推进。畅通政风行风热线，设立网络发言人，开展网络问政。强化行政监察、审计监督，“阳光村务工程”扎实推进，政风建设取得新成效。

  各位代表，2010年各项目标任务的顺利完成，为“十一五”画上了一个圆满的句号。五年来，我们一步一步打基础，一年一年增后劲，成功克服了自然灾害、金融危机等不利因素的影响，有力促进了经济社会持续快速发展。全市生产总值五年连跨三个百亿元台阶，年均增长11.5%左右，高于“十五”3个百分点以上；三次产业结构由40.5:24:35.5调整到26.7:37.8:35.5，二产上升13.8个百分点。财政收入连跨三个10亿元台阶，年均增长24.9%，高于“十五”13.4个百分点。固定资产累计投资突破千亿元，是“十五”的3.8倍，年均增长36.3%，建成了一批事关全局和长远的重大项目。中心城市建成区面积由30扩大到50平方公里，城镇化率五年提高10个百分点。累计引进内资855亿元，总量居皖北六市第一，实际利用外资4亿美元，年均分别增长46%、34.5%。城乡居民收入年均分别增长13%、15%以上。连续四年实施民生工程，累计投入财政资金65.6亿元，惠及500多万城乡居民。

  各位代表，“十一五”的五年，是我市砥砺奋进、艰苦创业的五年，是科学发展、加速崛起的五年，是综合实力提升最快、城乡面貌变化最大、人民群众受益最多的五年。成绩来之不易，经验弥足珍贵。我们深切体会到，在宿州这样一个后发地区，要实现经济社会又好又快发展，必须坚持把解放思想作为发展之先，锐意改革、开拓创新，积极探索符合市情的发展道路；必须坚持把发展作为第一要务，全力实施三大战略，以工业扩张锻造脊梁，以农业提升夯实基础，以城镇扩容提供支撑；必须坚持把扩大开放、加大投入作为推动发展的主抓手，多做打基础、管长远的工作，集聚发展要素，积蓄发展后劲；必须坚持把改善民生作为发展之本，多办利民之事，善解群众之忧，凝聚发展合力，营造加速崛起的浓厚氛围。

  各位代表，“十一五”的辉煌成就，主要归功于省委、省政府和市委的正确领导，归功于历届领导班子打下的坚实基础，归功于630万宿州人民的合力奋进！在此，我谨代表市人民政府，向全市广大干部群众，向各民主党派、工商联和人民团体，向驻宿解放军、武警官兵和政法干警，致以崇高的敬意！向关心、支持宿州发展的中央、省驻宿单位和海内外友好人士，向在我市创业的投资者、建设者，表示衷心的感谢！

  各位代表，回顾过去，我们也清醒地认识到，前进的道路上仍存在不少困难和问题，主要表现为：总量不大，结构不优，速度不快，与奋力赶超的要求还有不小差距；社会事业发展相对滞后，少部分群众生活困难，与兴宿富民的要求还有不小差距；少数干部思想不够解放，精神不够振奋，与加速崛起的要求还有不小差距；发展环境不优，工作效率不高，与开放发展的要求还有不小差距。对此，我们必须在今后发展中认真加以解决。

  二、继往开来，推动“十二五”加速崛起

  未来五年，是宿州大有可为的黄金发展期。纵观全局，国家中部崛起战略全面推进，东部地区产业转移加速，我省支持皖北振兴力度前所未有，宿州加速崛起的机遇十分难得。环顾四周，区域竞争日趋激烈，各地竞相发展态势逼人，小进势必垫底，大进才有出路，宿州追赶跨越正处在紧要关口。审视自身，我市发展基础不断巩固，区位优势日益彰显，投入效应持续释放，宿州奋力起跳条件具备。在这个重要节点，我们既耽误不得，更失误不起，唯有牢固树立奋勇争先的强烈意识，殚精竭虑，心无旁骛，方能不辱使命，不负重托！

  “十二五”经济社会发展的指导思想是：以邓小平理论和“三个代表”重要思想为指导，深入贯彻落实科学发展观，牢牢抓住产业转移、加快皖北发展的重大战略机遇，以转型发展、加速崛起、兴宿富民为主线，坚持工业化、城镇化双轮驱动，实施工业扩张、农业提升、城镇扩容战略，推动开放发展、创新发展、持续发展、和谐发展，实现国内生产总值、财政收入、固定资产投资、城乡居民收入翻番，打造实力宿州、活力宿州、和谐宿州、幸福宿州，力争走在皖北地区崛起前列，为全面建设小康社会奠定坚实基础。

  按照“总量进位次，人均上水平，缩小相对差，提高贡献率”的原则，综合考虑，在2013年完成“双千亿、双翻番”目标的基础上，力争到2015年，全市生产总值达到1300亿元，年均增长13%以上；财政收入达到130亿元，年均增长25%以上；固定资产投资年均增长25%以上；城乡居民收入年均增长13%以上，主要经济指标增速居皖北六市前列，进一步缩小与全省平均水平差距。

  “十二五”经济社会发展的主要任务是：

  ——大力实施工业扩张战略，构建现代产业体系。实施“7233”工程，以一区两园为载体，围绕实现700亿元工业增加值，打造煤电化、轻纺食品两个千亿元级产业基地，形成300亿元产值的战略性新兴产业集群，亿元以上产值企业超过300家，使工业经济成为加速崛起的主动力。按照“挖潜增效、培育支柱、完善功能、优化环境”的总体思路，把省级开发区建设成为工业发展主阵地，把新型乡村工业园建成农民创业的乐园。把推动服务业大发展作为产业结构调整的战略重点，推动商贸物流业、金融服务业、休闲旅游业、信息服务业等实现突破，带动现代服务业大发展、大繁荣。

  ——大力实施农业提升战略，推进社会主义新农村建设。实施“4168”工程，加快构建现代农业产业体系，推动农业大市向农业强市跨越。拓宽农民增收渠道，大力发展农村二、三产业，强力推进农业产业化经营，力争市级以上龙头企业达到350家，超亿元企业达到120家，农产品加工产值突破700亿元。加强农业基础设施建设，大幅度增加农业投入，改善农业生产条件，提高农业综合生产能力。创新农村发展体制机制，加快社会主义新农村建设，加快城乡发展一体化进程，建设农民幸福生活美好家园。

  ——大力实施城镇扩容战略，构建新型城镇体系。坚持中心城市与县域互动发展，城镇化与新农村建设同步推进，促进城乡规划、产业布局、基础设施和社会管理一体化。围绕建设皖东北中心城市，坚持片区开发、市区联动，推进汴河、城南、城东及老城四大片区建设，启动符离、蒿沟、朱仙庄、蕲县等经济功能区和卫星城镇建设，打造宜居宜业新宿城。加快县城和小城镇建设，形成以市区为核心、以县城为骨干、以重点镇为节点，功能定位清晰、片区布局合理的城镇体系。通过五年努力，中心城区超“双75”，四个县城超“双25”，城镇化率达到45%以上，提升城镇对发展的承载力和带动力。

  ——加快改革开放，增强经济发展活力。继续推动改革开放，努力在更大范围、更深层面集聚要素。加快行政管理体制改革，深化国有资产改革，推进事业单位改革，推进投融资体制改革，推进文化、医疗、教育体制改革，加快建立惠及全民的基本公共服务体系。加快构建地方金融体系，增强金融支撑作用。强化开放合作意识，全面加入长三角区域合作机制。准确把握新一轮产业转移规律，探索科学承接新模式，优化投资环境，提升招商引资质量和水平，力争五年累计到位内资突破3000亿元、外资10亿美元。

  ——转变经济发展方式，促进可持续发展。坚持绿色发展、低碳发展，加快形成资源节约、环境友好的生产方式和消费模式，全面完成节能减排省控目标。加快传统产业升级，推进高碳产业低碳化改造，深化重点技术攻关，构建循环产业体系。加大生态建设和环境保护力度，强化能源、土地、水、矿产等资源有序开发和集约利用，加快淘汰落后产能，减少主要污染物排放，切实提高可持续发展能力。推进科技创新和人才开发，完善以企业为主体、市场为导向、产学研相结合的技术创新体系，培育具有区域竞争力的人才优势，牢牢把握发展主动权。

  ——坚持民生优先，构建和谐宿州。把以人为本、民生优先的理念贯穿于经济社会发展全过程，实施创业就业、卫生健康、安居宜居、社会保障、平安祥和、和谐村居等六项民生幸福工程，努力实现人民群众学有所教、劳有所得、病有所医、老有所养、住有所居。支持全民创业，创造条件增加城乡居民收入，切实提高低收入群体收入水平。优先发展教育事业，优化教育资源布局，促进各类教育均衡发展。繁荣文化事业，大力发展文化产业，增强发展软实力。建立健全党委领导、政府负责、社会协同、公众参与的社会管理格局，强化安全发展理念，加强社会建设，巩固社会和谐稳定局面。

  三、着力开局，扎实做好2011年工作

  今年是实施“十二五”规划的开局之年，综合考虑各种因素，经济社会发展主要预期目标是：全市生产总值增长13.5%；财政收入增长高于生产总值增速；固定资产投资增长25%；社会消费品零售总额增长18%；城乡居民收入均增长13%以上；人口出生率控制在13.5％。以内；城镇登记失业率控制在4.5%以内；单位生产总值能耗和污染物排放达到省控目标。

  围绕上述目标，我们将重点抓好以下工作：

  （一）强力实施工业突破，加速壮大产业支撑

  继续推进工业五年扩张，以项目建设为抓手，确保工业投资增长35%，规模以上工业增加值增长25%以上。

  培育壮大主导产业。以实施重点产业规划为抓手，培育优势特色产业集群。全力推进在手重大项目，力促光大生物质能发电、格微光电、永通电缆、正冠陶瓷建成投产，加快海螺水泥、冠军陶瓷、元邦金属、国电宿州热电联产、金黄庄、龙王庙煤矿等项目建设步伐，推进骑路孙、张大屯、祁南二矿、朱楼煤矿尽早开工。加强与大型煤炭企业的战略合作，力争在煤化工产业上取得实质进展。开工建设220千伏宿南输变电工程。围绕国家政策支持重点，扶持发展节能环保、碳纤维等战略性新兴产业，推进产业优化升级。着眼主导产业延伸配套，研究、谋划、储备一批牵动性强的工业项目，特别是超10亿元的大项目，努力形成持续的投资后劲。

  做大做强现有企业。实施巨人培育计划，以100户成长性企业和150户重点调度企业为载体，在资金、财税、项目等方面倾斜，确保亿元以上企业超过150家。实施雏鹰成长计划，重点选择一批发展前景好的中小企业，加强信用担保和创业辅导，力争新增规模企业50家。继续实施骨干企业三年技改滚动计划，重点推进30个技改项目。加强产学研合作，重点扶持8家骨干企业建立研发中心，开工建设科技企业创业中心。加强20户上市后备企业培育，确保皖北药业上市。加强工业经济调度监测，强化要素支撑，促进增产增效。

  强力推动园区建设。推进园区产业集聚发展、集约发展、配套发展，加快建成工业新城和居住新区。加快园区基础设施建设，推进园区公共资源低成本共享。全力支持市经济开发区转型升级，启动新一轮规划修编，扩大园区面积，优化功能定位。规划建设10平方公里300亿元产值的制鞋基地，吸引大企业入驻，加快打造中部鞋都；加快推进皖北药业、中元化工、安特生化、惠丰电源、雪龙纤维等项目退城进园，入驻化工园区；尽快完成商贸物流园、不锈钢产业园、新兴产业园功能分区，明确发展目标，增强大项目落户承载力。积极吸引省内大企业筹建产业园区，合作共建宿州马鞍山工业园区。

  大力实施招商引资。抢抓新一轮产业转移机遇，建立政府引导、市场主导、企业为主的招商机制，力争全年到位内资突破360亿元、外资1.8亿美元。建立科学的业绩考核和经费保障机制，落实招商责任，提高考核权重，加大奖惩力度。增强招商实效，发挥企业主体作用，突出产业招商、专业招商、驻点招商。注重招大引强，紧盯500强企业搞好推介，深化与央企、省企、知名民企的合作，探索建立合作长效机制。进一步优化招商结构，加大农业、基础设施、文化、旅游、商贸等项目招商力度。加强招商项目落地服务，不断提高项目履约率和资金到位率。

  坚决抓好节能减排。严格落实节能减排责任制，推进节能减排全民行动。健全能耗监管机制，加快节能技术改造，做好重点企业节能工作。加快城镇污水管网建设，提高污水处理厂负荷和管网覆盖率，推进县级污水处理厂二期建设，在重点乡镇建设污水处理设施。加快市医疗废物集中处置中心和生活垃圾处理场建设。继续开展环保专项行动，严厉查处各类环境违法行为，坚决关闭“十五小”企业。加强流域污染防治，促进岱河、利民河、小黄河等河流水质明显改善。推进十镇百村千户生态示范建设，着力解决农村面源污染等突出环境问题。

  （二）强化中心城市带动，提升城镇化水平

  坚持城镇化适度超前，加快城乡基础设施建设，城镇化率提高2个百分点。

  加速中心城市建设。按照传承历史、面向未来、彰显文化、充满活力的要求，打造宜居宜业宜游和谐的新宿州。坚持规划引领，完成城市总规等16项规划编制任务，实现重点片区控详规全覆盖。重点实施路网、园林景观、公共公益设施、保障性住房和旧城改造五大类200项建设工程，确保总投资超过100亿元，进一步拉开城市框架，增强城市魅力与活力。强化运营城市理念，加大建设融资力度，尽快形成多元化投融资体制。坚持建管并重，巩固文明创建成果，推进土地征用、城市拆迁、市容管理等重心下移，强化区、街道和社区职能，落实城市管理责任。启动创建国家级园林城市工作，提升城市形象。引导广大市民充分参与到城市建设管理中来，在城市发展中获得更多实惠。

  加强城镇体系建设。全面完成各级土地利用总体规划修编、第二轮矿产资源规划编制。修编四个县城总体规划、中心镇总体规划，基本完成乡和划定区域内村庄规划调整，积极推进各专项规划修编。拓宽城镇建设融资渠道，每个县城建设投入不低于30亿元，推动县城建设提速。砀城要全力攻坚，推动四区一体联动发展，打造外圆内方、生态宜居的皖北水城；萧城要加快绕城线建设，加速融入徐州都市圈，实现环凤山发展；灵城要着眼灵璧石文化挖掘，提升内涵，打造现代山水园林县城；泗城要以创建省级文明县城为目标，突出生态宜居主题，打造独具特色的“水韵泗州”。支持有条件的重点镇按小城市标准规划建设，加速人口向城镇集聚。

  加快构筑现代综合交通运输体系。开工建设郑徐客运专线，推进徐明高速、泗许高速二期、济祁高速公路建设，力争宿淮铁路年底通车。加快新汴河河道及船闸治理工程报批。加快干线公路升级改造，启动建设山闵路、高铁站连接线、206国道改线工程，整治104、101等八条国省道，完成县道升级改造、通乡公路、连通工程等2400公里。强化县乡村三级公路管护。发展城市公共交通，构建农村客运网络，加强运输场站建设，实现各种运输方式有效衔接。

  （三）加快发展现代农业，大力促进强农富农

  把“三农”问题作为重中之重，围绕保障农产品供给、农民增收，强化农业基础，加快城乡一体化进程。

  加快推进农业转型升级。全面实施小麦高产攻关和玉米振兴计划，力争核心示范区单产突破千斤，粮食总产突破80亿斤。启动新一轮“菜篮子”工程，提高设施蔬菜生产水平，确保市场供应。加快发展规模养殖，力争规模化养殖比重达到54%。大力发展现代农业，主攻粮食等四大支柱产业，创新体制机制，建设农业支撑体系，扎实推进桥区国家级现代农业示范区建设，支持各县申报省级现代农业示范区。强化农业产业化龙头企业带动作用，加快标准化基地建设，农产品加工产值达到360亿元，农民专业合作组织发展到1600个，名优农产品品牌达到200个。

  改善农村生产生活条件。巩固农田水利基本建设成果，建立管护长效机制，完成易涝农田治理97万亩、农业综合开发土地治理10.5万亩，建设高标准良田9万亩。加快推进小型农田水利重点县和专项县建设任务，完成文家河、利民河、龙岱河、洪碱河、屎印⒗虾缌楣怠⒚窭河治理任务，完成3座病险水库除险加固、新汴河灌区续建配套与节水改造工程。进一步提高大型农机化装备水平。加快改造农村公路危桥。继续实施万里绿色长廊、长防林、退耕还林等工程，完成人工造林11.2万亩，封山育林4.8万亩，增加森林碳汇。推进教育、文化、医疗等公益事业向农村延伸，再解决51.9万农村人口饮水安全问题。多渠道促进农民增收，加快发展农村二、三产业，全面拓宽非农就业空间，增加农民家庭经营性和财产性收入。认真落实各项强农惠农政策，让农民得到更多实惠。

  （四）努力提升第三产业，保持消费需求持续增长

  积极落实扩大消费需求方针，推动服务业发展提速、结构提升，力争服务业增加值占GDP比重提高2个百分点。

  推进市场体系和物流基地建设。加快综合市场、专业市场、配送中心等城乡市场体系建设，重点培育奇石、蔬菜批发、粮食市场，推进合百大农产品物流、白马商城等项目建设，形成一定规模的现代市场集群。加快西南片区、高铁站前广场等物流和仓储园区建设，规划建设屏山等公路铁路货运站，培育发展一批大中型现代物流企业。加强政策性支持，推进传统批发经营形式创新，努力形成大市场、大流通格局。

  加快发展现代服务业。提升中心城区商业功能，加快皖煤、宿州国际两个五星级酒店建设，促进华夏世贸、家乐福等早日投入使用，形成中心商圈。着力发展餐饮、购物、农贸等生活性服务业，扩大文化、休闲、健身等服务性消费，促进传统业态创新。加快发展中介、保险、法律咨询等现代服务业，积极培育汽车、住房、社区服务等消费热点。健全农村消费品流通体系，继续推进“万村千乡”市场工程建设，做好汽车摩托车下乡、家电下乡及以旧换新工作，扩大农家店经营范围，建设一批乡镇直营店，充分释放农村消费市场潜力。组建村镇银行、贷款公司、资金互助社等新型农村金融机构到县，设立灵璧农村银行，支持徽商银行扩大网络，争取马鞍山农村商业银行等设点落户，不断增强对中小企业的担保能力。优化金融生态环境，深入推进金融产品和服务创新，综合运用考核激励等手段，引导金融机构扩大信贷投入。

  壮大旅游经济实力。认真实施旅游业发展规划，建设旅游精品，打造旅游品牌。统筹整合自然、历史、人文等旅游资源，大力发展生态旅游、红色旅游、文化旅游，着力打造以皇藏峪——黄河故道为代表的生态旅游、以楚汉文化为代表的文化旅游精品路线，支持灵璧县申报灵璧石国家级地质公园，重点开发虞姬文化园、五柳、蔡洼、石龙湖和岳庄坝湿地公园等精品景点。注重景区文化内涵包装宣传，加大旅游促销力度，促进城乡旅游联动发展。

  （五）深入推进改革开放，加速释放发展活力

  以更大力度深化改革、扩大开放，进一步集聚发展动力，不断提高开放型经济水平。

  继续深化各项改革。大力发展非公经济，进一步降低准入门槛，鼓励引导民间投资，激发民间资本活力。继续抓好国有企业改制。推进事业单位分类改革工作。完善财政预算体系，深化政府采购改革，推行预算绩效评价试点。巩固基层医药卫生体制综合改革成果，推行村卫生室一体化管理，加大乡镇卫生院绩效考核。推进公立医院改革试点，优化调整公立医院区域布局结构。深化农村综合改革，积极稳妥推进土地承包经营权流转、水管体制、集体林权制度改革，扩大一事一议财政奖补试点范围，完成基层农业技术推广体系改革。

  不断扩大对外开放。积极参与泛长三角区域、黄淮海区域合作分工，全方位开展与马鞍山对接交流，不断提升合作层次和水平。高水平办好黄淮海地区农资博览会、砀山梨花旅游节、泗州戏艺术节等活动，推动会展活动向会展经济发展。积极参加各类经贸活动，重点举办面向长三角等地区的投资推介活动。强化对企业的跟踪服务，尽力满足项目建设和企业发展需求，努力营造开明开放、诚信重诺、宜居宜业的发展环境。进一步加大进出口企业、基地和集群扶持力度，培育壮大出口主体，不断提升经济外向度。

  （六）全力振兴县域经济，推进区域协调发展

  突出县域经济在全市发展格局中的战略地位，加大支持力度，让县域经济成为宿州加速崛起的重要支撑。

  加快打造产业特色。加大县域产业结构调整力度，形成各具特色的产业集群。坚持分类指导，发挥资源优势，支持砀山加速培育果蔬加工等四个百亿元产业，打造全国水果加工第一大县；支持萧县大力发展矿产、陶瓷等四大支柱产业，在全市实现率先争先目标；支持灵璧实施“1356”行动计划，力争跻身皖北县域发展第一方阵；支持泗县东向发展，打造纺织服装、农副产品加工双百亿产业；支持桥区依托“四园两带”，提高首位度，争当排头兵。全力推进各县区开发区建设，引导各类要素向园区集中，促进关联产业聚集，实现错位发展。加快乡村工业园建设，支持周寨、草沟、符离、下楼、城北等重点乡镇园区早日跻身省级开发区。开展扩权强镇试点，培育一批工业增加值超亿元、财政收入过千万元的乡镇。

  持续加大政策扶持。按照标杆引领、联动发展的原则，确定每个县区对标县，借鉴苏北“洼地崛起”的经验，争先进位，确保县域经济发展速度超过全市平均水平。完善县域经济综合考核体系，把跟跑对标县的成效列入考核范畴，定期观摩调度重点项目建设，促进县域经济竞相发展。全面落实支持皖北发展政策，放足、放活市级审批权限，进一步强化要素支持，改进县域金融服务，强力推进城乡建设用地增减挂钩，完成农村土地整治5万亩，积极为县区争取用地指标、点供计划。严格用地预审和土地供应，加大闲置土地清理力度。各县区要始终保持不甘落后、奋发有为的精神状态，激发内生活力，在宿州加速崛起进程中挑重担、扛大梁。

  （七）全面发展社会事业，维护和谐稳定局面

  不断加大社会事业投入，努力为全市人民提供更多、更优质的公共产品和社会服务。

  协调发展各项社会事业。加快构建公共文化服务体系，抓好文化惠民工程。加强优秀文化资源挖掘和保护，推动文化产业园建设，培育文化创意、文化旅游等新兴文化产业。办好市第四届体育运动会。千方百计稳定低生育水平，加强计划生育服务体系建设，遏制出生人口性别比偏高势头。全面落实各项人才政策，启动市级人力资源市场建设。坚持社会主义核心价值体系，巩固精神文明建设成果。依法加强民族宗教事务管理。加强国防动员、人防和民兵预备役建设，争创全国双拥模范城六连冠。拓展城市社区服务，提高社会化服务水平。做好第八届村民委员会换届选举工作。启动实施“六五”普法，建立法律援助财政保障机制。支持工会、共青团、妇联开展工作。积极推动防震减灾、扶贫开发、外事侨务、对台、烟草、气象、档案、地方志、红十字、老龄、残疾人等事业发展。

  切实维护社会稳定。高度重视物价上涨对群众生活的影响，关注低收入群体，全面加强价格调控监管工作。大力推进平安宿州建设，构建社会治安综合防控体系，深化“三级巡控”机制，严厉打击违法犯罪。加强和改进信访工作，完善社会矛盾纠纷排查调处机制，着力解决信访突出问题。健全安全生产监管机制，强化企业主体责任，突出抓好重点领域安全监管，保持道路交通和火灾预防高压态势，坚决杜绝重特大事故发生。提高产品质量和食品药品安全监管水平。完善应急管理体系，提高应对突发事件处置能力。

  （八）高度关注民生幸福，着力解决群众关心的利益问题

  为人民群众做实事、办好事、解难事是各级政府的责任。今年，市政府将重点围绕六项民生幸福工程，继续抓好33项民生工程建设，致力民生改善。

  学有所教。一是努力解决农民工子女和留守儿童就学问题，资助中职学校贫困学生，决不让孩子因贫失学。二是实施学前教育三年行动计划，力争学前一年入园率达85%以上，学前三年入园率达65%以上。三是调整优化中小学校布局，推进义务教育阶段学校标准化建设，提升农村学校和薄弱学校办学水平，确保西北新区中学今年秋季招生。四是投入11亿元，加固重建校舍安全工程150万平方米。五是支持宿州学院发展，筹建泗州戏学院，推进宿州职业技术学院创建省级示范高职学院，促进宿州卫校早日升格。六是突出发展职业教育，推动宿州技师学院新体制运转，组建中职教育集团，启动宿州职教园区建设。

  病有所医。一是扩大基本医疗保障覆盖面，力争参合率达到95%以上，让多数百姓都能享受到医疗保障的阳光。二是提高新农合保障水平，全面实行门诊统筹，住院费用报销比例提高到60%。三是投入使用市立医院、皖北总院医疗综合楼，加快市中医院、精神病院、传染病医院、开发区医院建设，加强县以下卫生服务机构规范化建设。四是免费提供9项基本公共卫生均等化服务，实行农村孕产妇免费住院分娩。

  劳有所得。一是加强技能培训，加快建设农民工返乡创业园，促进农民就地就业。二是重点面向大中专和中职院校毕业生、农民工和下岗失业人员，组织开展就业援助活动。三是建立建筑领域农民工工资拖欠单位信用评价制度，在施工企业全面推行打卡发薪制度。四是建立企业职工收入和离退休人员养老金合理增长的长效机制，提高低收入群体收入水平。

  老有所养。今年市财政安排社会保障方面的支出7.5亿元。一是扩大社会保障覆盖面，将未参保集体企业退休人员全部纳入基本养老保险。二是巩固完善基本养老保险和城镇居民基本医疗保险市级统筹，实行失业保险、工伤保险市级统筹，推动城镇职工基本医疗保险市级统筹，完善城镇职工基本医疗保险费用结算办法。三是落实被征地农民社会保障措施，建立农村特殊群体养老保险制度，积极开展新型农村养老保险试点工作。

  住有所居。一是扩大廉租住房补贴面，保障户数1.4万户，建设廉租房2110套、10万平方米。二是建设公共租赁住房590套。三是新建经济适用房3.2万平方米。四是完成棚户区改造9800户。五是加强房地产市场监管，发挥住房公积金作用，努力增加中低价位、中小户型商品房供给总量。

  市政府在广泛征求意见的基础上，决定实施9件惠民实事。

  1．启动建设市体育馆。

  2．启动建设市文化艺术中心（大剧院）。

  3．启动建设市残疾人康复中心。

  4．实施城区集中供热工程，完成主管网建设16.7公里。

  5．加快供水管网改造，市区自来水力争实现一户一表。

  6．加快老城改造，全市新建安置房和保障性住房400万平方米，其中市区150万平方米。

  7．市区新建改造5个菜市场。

  8．新增蔬菜基地12.3万亩，其中市区新建1万亩设施蔬菜栽培基地。

  9．新建扩建27所农村规范化敬老院，新增床位1800张。

  四、勇于担当，全力打造人民满意政府

  面对新的形势、新的任务，各级政府及其工作人员必须始终把对人民负责、让人民满意作为政府工作的第一准则、第一追求，努力塑造负责任、敢担当、干实事、有作为的崭新形象。

  让人民满意，必须依法行政，规范作为，建设法治政府。自觉按照法定权限和程序行使权力、履行职责，依法、民主、科学决策，让政府权力在阳光下运行。自觉接受人大及其常委会的法律监督，认真执行决议，定期报告工作，积极支持政协履行政治协商、民主监督和参政议政职能，认真办理议案、建议、提案。加强行政执法队伍建设，规范行政执法行为，促进严格、公正、文明执法。

  让人民满意，必须脚踏实地，亲民爱民，建设为民政府。时刻牢记为民宗旨，自觉做到“一枝一叶总关情”。坚持公共财政向困难群众、农村、基层和公共事业倾斜，解决好最困难群众最需要解决的突出问题。加快电子政务建设，充分发挥市长热线、民生热线、信访网络的桥梁纽带作用，最大程度地贴近群众，倾听群众呼声，切实为群众解难题、办实事。深入基层和工作一线，积极为群众、为企业排忧解难，切实营造人民满意的政务服务环境。

  让人民满意，必须开拓创新，优质高效，建设效能政府。狠抓政风行风建设，推动工作落实。要以目标管理总揽全局，强化任务分解，严格督查考核，落实责任主体，坚决兑现奖惩，形成一级抓一级、一级对一级负责的责任落实机制。加强行政服务中心建设，深化行政审批制度改革，推进政府职能转变，促进机关服务提速增效。扎实开展效能监察，加大纠风治乱力度，严查不作为、慢作为、乱作为，打造经济发展软环境。深入开展争创“四型”机关、争做优秀公仆活动，以清新的政风凝聚民心。

  让人民满意，必须反腐倡廉，勤俭节约，建设廉洁政府。坚持从严治政，强化对重要领域、关键岗位的行政监察和审计监督，认真治理工程建设领域的突出问题，切实加强国有资产和工程招投标、政府采购、土地出让等重点领域的监管，坚决纠正损害群众利益的不正之风。牢固树立过紧日子的思想，坚持勤俭办事业，反对奢侈浪费。加强廉政文化建设和反腐倡廉制度建设，加大从源头预防和治理腐败的力度，坚决查处各类违法违纪行为。

  各位代表！人民的重托千钧在肩，发展的使命任重道远，时不我待，只争朝夕。让我们紧密团结在以胡锦涛同志为总书记的党中央周围，深入贯彻落实科学发展观，在中共宿州市委的坚强领导下，振奋精神，凝心聚力，埋头苦干，开拓奋进，为加速建成四省交汇的区域中心城市，开创宿州的美好未来而努力奋斗！
